# Motion compensated micro-CT reconstruction for in-situ analysis of dynamic processes

**DOI:** 10.1038/s41598-018-25916-5

**Published:** 2018-05-16

**Authors:** Thomas De Schryver, Manuel Dierick, Marjolein Heyndrickx, Jeroen Van Stappen, Marijn A. Boone, Luc Van Hoorebeke, Matthieu N. Boone

**Affiliations:** 1 Radiation Physics research group, Dept. Physics and Astronomy, Ghent University, Proeftuinstraat 86/N12, 9000 Gent, Belgium; 2 PProGRess research group, Dept. Geology, Ghent University, Krijgslaan 281/S8, 9000 Gent, Belgium; 3 Ghent University Centre for X-ray Tomography (UGCT), Proeftuinstraat 86, 9000 Gent, Belgium; 4 Present Address: XRE NV, Bollebergen 2B/1, 9052 Gent, Belgium

## Abstract

This work presents a framework to exploit the synergy between Digital Volume Correlation (DVC) and iterative CT reconstruction to enhance the quality of high-resolution dynamic X-ray CT (4D-µCT) and obtain quantitative results from the acquired dataset in the form of 3D strain maps which can be directly correlated to the material properties. Furthermore, we show that the developed framework is capable of strongly reducing motion artifacts even in a dataset containing a single 360° rotation.

## Introduction

High-resolution X-ray Computed Tomography or *μ*CT is a valuable non-destructive 3D imaging technique in numerous research areas. It has matured to the point where it is now possible to image dynamic processes within micro-structures in 3D with a sufficient temporal resolution to follow the microstructural changes caused by these process through time, i.e. in 4D (e.g.^1^). Especially synchrotron experiments, armed with the luxury of high X-ray flux, have pushed the boundaries of the temporal resolution up to the 1 ms range, at spatial resolution as good as 3 *μ*m^2,3^. An exhaustive survey on the achievements w.r.t. temporal and spatial resolutions, can be found in literature^4,5^, from which it should be noted that high temporal/spatial resolution CT imaging is becoming tractable at lab-based CT facilities as well^6,7^. Though compared with synchrotron sources they are advantageous in terms of system cost and offer the freedom to move the X-ray source, the major limitation for the lab-based setups is the limited X-ray flux produced by micro-focused X-ray tubes. This subsequently limits the temporal resolution due to a lack of photon statistics at the lowest detector exposure times. Hence, to reach respectable temporal resolutions in a lab-based setup, a trade-off between an acceptable level of noise and motion blurring has to be made. In general, motion blurring can be caused by any structural change following a dynamic process within the sample, e.g. stress induced internal deformations, temperature related phase changes, imbibition of fluids in micro-pores etc. Although motion blurring is mainly caused by the discretization of the process in a finite number of time frames rather than the speed of the process, it is of course aggravated in fast processes where increasing the number of frames is no longer possible due to hardware limitations or the extreme amount of shot noise in the images. To further boost the temporal frequencies of CT imaging, a priori information on the underlying dynamic processes can be incorporated in the CT reconstruction algorithm, in most cases of the iterative type. The precise nature of this a priori information, which strongly depends on the imaged process, has to be cast in a mathematical formulation which models the interaction between the dynamics of the process and the imaged quantity, here the X-ray attenuation coefficient. The concept is nicely illustrated by Myers and co-authors^8,9^, where the a priori information is meant to model fluid displacements in the rigid matrix of a porous rocks. In our work however, we consider a dynamic non-rigid deformation of the sample’s micro-structure. An estimate of this local deformation could serve as a priori knowledge to compensate for motion blurring upon its integration in a CT reconstruction algorithm. To achieve this (1) a dense displacement field has to be estimated, based on a dynamic CT acquisition and (2) the projection and back-projection steps in the reconstruction algorithm have to be altered in order to account for a deforming image volume.

Estimating a displacement field in 3D is commonly referred to as Digital Volume Correlation (DVC), which optimises the correlation between a deformed volume and a reference volume by mapping the former to the latter through a motion vector field (MVF). The ability to calculate a full-field strain mapping from this MVF, through proper differentiation, has led to an increasing interest for DVC in the material research community ever since its introduction^10,11^. Particularly in the field of biomechanics, deformations in trabecular bone have been studied intensively using DVC^10,12^, but also in other material disciplines like wood^13,14^, metal foam^15–17^, gypsum^18^, soil^19,20^, sand packings^21–23^ and composites^24^ DVC has been leveraged to produce strain maps.

While DVC has found its way to *μ*CT material research, its potential to compensate for motion blurring has not been given much attention, as in most applications both the reference and deformed images are derived from high quality static CT scans. However, when the dynamics of a deformation process are at the edge of a tractable temporal resolution for imaging, and are not controllable, i.e. there is no clear distinction between reference and deformed states, DVC and CT reconstruction can be integrated to enhance the quality of both, in terms of signal-to-noise ratio (SNR), temporal and spatial resolution. The idea to exploit this synergy between DVC and motion compensated CT reconstruction has been explored for medical CT several years ago^25–28^, and has recently also found its way into high-resolution X-ray CT^29,30^.

This work presents a framework to reconstruct datasets imaging a dynamic process, using one or more full rotations. Different DVC methods are compared. We illustrate the potential of this framework to reduce motion artefacts in a single rotation scan, and to combine this with the retrieval of the displacement field for the full object during multiple rotations. With a view to mapping the material properties in micro-structured materials, the displacement is referenced to the original volume and the concept is applied to a series of high-speed *μ*CT acquisitions of an *in-situ* aluminium foam compression experiment. More importantly, we show that the framework is capable of successfully compensating for motion artefacts, even in a conventional *μ*CT scan using only a single rotation where sample movement is unintended.

## Methods

### Digital Volume Correlation

During the registration of two images, a spatial transformation that optimises the similarity between these images is estimated. This similarity can be defined in several ways, but a plausible though rigorous approach in the context of registering CT attenuation images, is to instate the ‘*constancy of brightness*’:

1 $$\mu ({\overrightarrow{x}}_{m},\,{\tau }_{m})=\mu \,({\overrightarrow{x}}_{f}+\overrightarrow{u}({\overrightarrow{x}}_{f},\,{\tau }_{f}),\,{\tau }_{f}),$$

where $\mu (\bar{x},\,t)$ is the reconstructed attenuation coefficient at position $\bar{x}$ in 3-dimensional space and at time point *t*, $\bar{u}(\bar{x},\,t)$ is the displacement of the voxel at postition $\bar{x}$ at time point t, and the indices *f* and *m* denote the fixed state and moving state, respectively. The indices will be omitted in the following for notational simplicity, as *t*_*f*_ can be set to zero and we will always refer to the fixed position: $\bar{x}={\bar{x}}_{f}$ and ${\bar{x}}_{m}={\bar{x}}_{f}+\bar{u}(\bar{x},\,t)$. In (1), the images are implicitely linked to distinct instances in time (*t*_*f*_ and *t*_*m*_), which underlines the fact that in order to match the fixed and moving states, a time evolving deformation field $\bar{u}({\bar{x}}_{f},\,{t}_{m})$ is to be inferred from the time evolving attenuation distribution of the object. The goal is thus to find an MVF that deforms the fixed image (index *f*) such that it matches or in the ideal case is equal to the moving image (index *m*). When these images are represented by 3D volumes, image registration is typically referred to as *‘Digital Volume Correlation’* (DVC).

Following^31^, the essential components of any registration algorithm are (1) the choice on how the MVF is parametrised, (2) the metric used to quantify the similarity between the fixed and moving images, and (3) the strategy used to find an optimal set for the deformation parameters.

In this work, the entire MVF is described as a linear combination of B-splines, set out on a regularly spaced grid of control points. In 3D, the displacement component can thus be approximated by

2 $${u}_{(x,y,z)}(\bar{x})=\sum _{{n}_{x},{n}_{y},{n}_{z}=1}^{{N}_{x},{N}_{y},{N}_{z}}{w}_{(x,y,z)}({n}_{x},\,{n}_{y},\,{n}_{z})\,{ {\mathcal B} }_{{n}_{x},k}(x)\,{ {\mathcal B} }_{{n}_{y},k}(y)\,{ {\mathcal B} }_{{n}_{z},k}(z)$$

where *k* indicates the B-spline’s order, *N*_*x*_ fixes the number of control points, and the *w*-values form a set of parameters that fix the motion model. In 3D, the linear expansion basis is constructed out of a B-spline tensor products, which are now used to approximate three displacement components,

The MVF is thus described by a parameter vector field $\bar{w}=[{w}_{x},\,{w}_{y},\,{w}_{z}]$. The ‘*elastix*’ toolbox^31^, which is also exposed to Python through the SimpleElastix module^32^, was used to optimise this parameter vector towards a maximalistion of the ‘*Normalised Cross Correlation*’ (NCC) metric with an ‘*Adaptive Stochastic Gradient Descent (ASGD)* optimiser^33^.

SimpleElastix is an extension to the SimpleITK module^34–36^, which also provides an implementation of B-spline registration and several other algorithms, such as the Demons registration approach^37^. This Demons approach directly enforces brightness constancy, i.e. Equation (1), by considering its Taylor expansion ${\mu }_{\tau }+\bar{u}\cdot {\nabla }_{\bar{x}}\mu =0$. The resulting optical flow constraint^38^ can also be formulated in an image’s phase domain, leading to yet another registration approach using dual-tree complex wavelets^39,40^. It is not the goal of this work to provide in depth mathematical explanation of the Demons and phase based approaches, for which the reader is referred to the above cited literature, and the short overview found in^41^. Furthermore, it will be shown that the B-spline approach is in fact the best choice for the application at hand, both in terms of registration quality and computational requirements.

### Motion corrected CT reconstruction

In 4D-*μ*CT imaging a delicate balance must be maintained between motion blurring artefacts, arising when a dynamic process is imaged at an insufficient temporal resolution, and a low SNR, as a result of acquiring an insufficient number of photons per projection during fast imaging. When considering non-rigidly deforming samples, both the motion blurring and low SNR can be alleviated by integrating an estimate of the deformation into the CT reconstruction algorithm. The following describes how the Simultaneous Algebraic Reconstruction Technique (SART)^42,43^ was adapted to incorporate a warp $\bar{u}(\bar{x},\,{t}_{p})$ into the projection and back-projection steps. Important to note here is that the warp is indeed a function of time, where *t*_*p*_ is the instant at which projection *p* is acquired. With a fixed exposure time *t*_*exp*_ for each projection, the timescale of a dynamic acquisition is thus set by *t*_*p*_ = *t*_0_ + *p*·*t*_*exp*_. In this way, a CT reconstruction reflects the state of an object at a time instant *τ* which can be approximated by averaging the time indices across the set *S*_*τ*_ of *N*_*τ*_ projections used to reconstruct the 3D volume (cfr.^44^):

3 $$\tau =\frac{1}{{N}_{\tau }}\sum _{p\in {S}_{\tau }}{t}_{p}$$

To reconstruct the time instant *τ*, the SART algorithm iteratively implements a sequence of three steps while looping (randomly or in a well-defined scheme) over each projection in *S*_*τ*_. First of all, a projection image ${\hat{P}}_{p}$ is simulated from the intermediate solution *μ*^(*n*)^ at iteration *n* by estimating the line integral of the X-ray attenuation coefficient along the rays which connect the detector pixel *j* to the X-ray source. At iteration *n*, this amounts to

4 $${\hat{P}}_{p,j}^{(n)}={l}_{j}\sum _{i\mathrm{=0}}L{\mu }^{(n)}({\bar{x}}_{ji})$$

where, following Joseph’s approach^45^, the *L* points ${\overrightarrow{x}}_{ji}$ are equidistantly sampled with an interval *l*_*j*_ on the ray that connects the source point ${\overrightarrow{O}}_{s}$ to detector pixel *j*. In the second step, the error with respect to the real measurement *P*_*r*, *j*_ is calculated and weighted by a factor containing the relaxation *λ*, a weight ${w}_{p}^{c}$ reflecting the contribution of projection *P*_*p*_ to the current time step *t*_*p*_, an aperture function ${w}_{j}^{a}$ to reduce artefacts inherent to region of interest reconstructions^26^, and the intersection length between the ray *j* and the voxel volume.

5 $${C}_{p,\,j}^{(n)}=({P}_{p,j}-{\hat{P}}_{p,j}^{(n)})\cdot \frac{\lambda {w}_{p}^{c}\,{w}_{j}^{a}}{L{l}_{j}}$$

Finally, during the back projection step, the *μ*-value in each voxel is updated by interpolating the correction term ${C}_{p}^{(n)}$ at the intersection between the detector plane and the line connecting the source positions ${\overrightarrow{O}}_{s}$ to the voxel’s position ${\bar{x}}_{m}$. The resulting interpolation value is then added to the previous estimate for the *μ*-distribution

6 $${\mu }^{(k+\mathrm{1)}}({\overrightarrow{x}}_{m})={\mu }^{(k)}({\overrightarrow{x}}_{m})+{C}^{(k)}({\overrightarrow{O}}_{s},\,{\bar{x}}_{m}).$$

The goal is now to go beyond the local time stationarity assumption, and adapt the reconstruction algorithm to cope with deformations (cfr.^25,26^). It is assumed that the deformation fields can be described by dense displacement fields ${\bar{u}}_{p}(\bar{x})$, known in advance^29^ or estimated through any registration technique. Important here is the indexation ‘*p*’, which indicates that the ${\bar{u}}_{p}$ describes the displacement of the object at time instant *t*_*p*_, when the projection *P*_*p*_ is acquired. Given this definition, the projection step (4) and back projection step (6) of the SART reconstruction algorithm can be adapted to:

7 $${\hat{P}}_{p,j}^{(n)}={l}_{j}\sum _{i\mathrm{=0}}L{\mu }^{(n)}({\bar{x}}_{ji}+{\bar{u}}_{p})$$

8 $${\mu }^{(k+\mathrm{1)}}({\bar{x}}_{m})={\mu }^{(k)}({\bar{x}}_{m})+{C}^{(k)}({\bar{O}}_{s},\,{\bar{x}}_{m}-{\bar{u}}_{p}({\bar{x}}_{m}\mathrm{))}.$$

Equations (7) and (8) also give some hints on how the motion correction can be efficiently implemented. In a first, brute force approach the deformation transform could be executed across the entire intermediate reconstruction volume. In handling a projection *P*_*p*_, the reconstruction volume is thus reverted to its state at that time. After the back projection, the volume is then deformed back to the reference time. This amounts to a twofold resampling of the entire volume, which for one, is a computationally demanding task, especially because it needs to be executed during each SART iteration, but foremost, might also induce an interpolation error to be carried and accumulated across the many SART iterations. In this work, a somewhat more lightweight implementation is considered, that uses a non-parametric displacement vector field as an overlay to the reconstruction volume. The major advantage of this approach is that the three displacement vector components (*u*_*x*_, *u*_*y*_ and *u*_*z*_) can be stored in the texture memory of a GPU. Using the texture’s built in trilinear interpolation capabilities, a displacement vector can be sampled, which is then used to redefine the position at which the *μ*-volume is sampled. While this approach introduces virtually no overhead with respect to the original SART implementation (more specifically only three texture memory fetches and three additions), there are some limitations to be considered:

- The approach requires more memory, as now three extra volumes need to be stored in GPU RAM. However, the displacement vector volumes do not have to be represented at the same resolution level as the *μ*-volume. A down-sampling can be carried out, of course at the cost of some loss in the displacement field’s accuracy.
- The parametrisation of the registration technique used to estimate the displacement vectors is lost. This does not mean that the registration procedure itself will not benefit from a more advanced parametric motion model. In the SART iterations, the vector field will simply not be sampled according to this motion model. A B-spline sampling model could be implemented on the GPU, but then the advantages of using textures will have to be sacrificed.
- The technique falls or stands with a good model for the displacement field’s evolution at each time point *t*_*p*_.

Indeed, this last remark is crucial, but since a registration procedure between a fixed image at time *t*_*f*_ and a moving image at *t*_*m*_ only yields a single displacement field without any information on its temporal information, not a lot of choices are left for the parametrisation of its temporal behaviour. In this work, we use a simple linear evolution of a displacement field

9 $${\bar{u}}_{p}(\bar{x})={\bar{u}}_{m}(\bar{x})\frac{{t}_{p}-{\tau }_{f}}{{\tau }_{m}-{\tau }_{f}}.$$

In practice, the linear evolution will be calculated between the projection timestamp *t*_*p*_ and the average time *τ* of the reconstruction set *S*_*τ*_. Although this linear interpolation already yields a great improvement on the reconstruction, as will be shown in the results, it is important to note that more complex functions can be introduced for 4D-*μ*CT datasets containing multiple frames. These complex functions will however increase the computational complexity and memory requirements.

### Evaluation parameter

For the evaluation of the registration, a scoring parameter *χ*_*s*_ is used, representing the ratio of two normalised cross correlations (NCC):

10 $${\chi }_{s}({n}_{v})=\frac{{\rm{NCC}}({\mu }_{m},\,{\mu }_{f}^{\ast })}{{\rm{NCC}}({\mu }_{m},\,{\mu }_{f})}-1$$

where *n*_*v*_ is the nominal displacement, μ* is the transformed fixed image ${\mu }_{f}^{\ast }(\bar{x})={\mu }_{f}(\bar{x}+\bar{u}(\bar{x}))$ and subscripts *f* and *m* again denote the fixed and the moving volume, respectively. The pretence of *χ*_*s*_ is to have an indication on how good the correspondence between the transformed fixed image and the moving image really is, i.e. if the registration technique effectively removes the mismatch between the original fixed image and the moving image, which is the case if *χ*_*s*_ > 0.

### Experimental data

The experimental data is acquired using the Environmental Micro-CT (EMCT) scanner of the Ghent University Centre for X-ray Tomography (UGCT)^6^. This unique gantry-based system allows to perform continuous (i.e. without rotating back to a reference position) CT scanning of an object which is kept stationary. This is ideal for objects inside peripheral equipment, limiting the rotational movement of the sample due to wires and tubes connected to it. Furthermore, this system is optimized for fast acquisition, reaching a full rotation in 12 seconds at a spatial resolution better than 20 *μ*m^5^. A CT5000 compression stage (Deben UK Ltd., Suffolck, UK) was installed at the EMCT system and used to compress aluminium foams at a controllable strain rate. The deformation of metal foams has been studied before with X-ray *μ*CT in literature^16,17^. Besides its use as an advanced engineering material^15^, due to its low weight an load bearing capacity, it has also been brought forward as a surrogate model for bone, which has a similar micro structure, though a rather brittle mechanical behaviour as opposed to the ductile nature of aluminium^11,46^. The precise properties of aluminium foams strongly depend on the way they are manufactured, leading to a wide variety in structures of an open- or closed-cell type, and with various aluminium strut thicknesses and air pore volumes^47^. The foam used here was an open cell porous aluminium foam (10 ppi, porosity ≈ 95%) provided by the Applied thermodynamics and heat transfer group at the UGent Department of Flow, Heat and Combustion mechanics, that studies these foams in their capacity to efficiently transfer heat^48^. With the goal of optimising this heat transfer the foams are manufactured to have a high surface area to volume ratio, resulting in relatively thin aluminium struts (approx. 200 *μ*m). Their behaviour is still fairly predictable, as they tend to deform in a smooth plastic way, with a small amount of discrete motion events, such as buckling or brittle strut breaking. This behavior makes these foams an ideal candidate to benchmark the registration approaches presented in this work.

A cubical aluminium foam sample, with a side length of approx. 1.5 cm, was placed in a cylindrical PEEK (Polyether ether ketone) container, which is attached to the bottom (moving) plate of the Deben compression cell. A piston, attached to the top (stationary) plate, inserts itself into the PEEK container, as the bottom part moves upward, thus effectuating a compressive action upon the aluminium foam cube.

In a first experiment, the acquisition was performed fast with respect to the compression speed in order to minimize the motion artefacts in a single rotation. In this experiment, the quality of the reconstruction can be assessed as a function of displacement. The compression rate was set to 0.5 mm/min, and 60 gantry rotations measuring 700 projections per rotation were completed during the 14 min acquisition at an exposure time of 20 ms. This amounts to a total compression of approx. 8 mm or approx. 133 *μ*m per rotation. Each of the rotations in this 34 GB dataset can be reconstructed separately, showing a relatively low degree of motion blurring. Due to the inherent contrast of the aluminium foam structure with respect to its air-filled background, the low SNR of 14 sec rotations was not an issue.

In a second experiment, our framework was evaluated as a tool to reduce motion artefacts in a single (360) rotation. To achieve this, the compression rate was fixed at 0.1 mm/min while imaging it with a standard single rotation cone beam acquisition using 1500 projections. Three different exposure times (160 ms, 320 ms and 640 ms) were selected, corresponding to a scanning time of 4, 8 and 16 minutes and a compression of 0.4 mm, 0.8 mm and 1.6 mm, respectively.

An overview of the scanner settings for both experiments is given in Table 1.

**Table 1:** Imaging parameters for the experimental data.

	Experiment 1	Experiment 2
Tube voltage (kV)	90	90
Tube power (W)	19.8	16
Detector pixel size (*μ*m)	200	200
Detector width & height (pixels)	658 × 656	658 × 656
Source-object distance (mm)	73.4	71.4
Source-detector distance (mm)	367.02	367.02
Magnified pixel pitch (*μ*m)	40	39

### Data availability statement

The data can be made available upon reasonable request.

## Results

### Varying displacement

**Figure 1:**
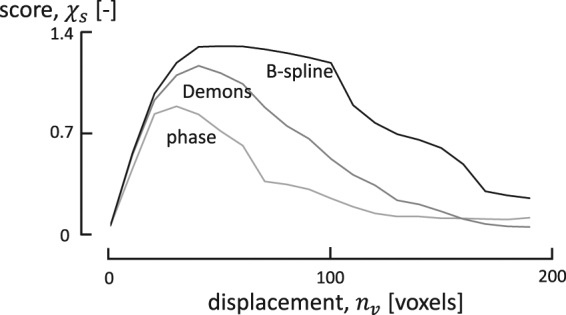
Scoring curves for Demons, phase flow and B-spline registration for the aluminium foam acquisition. The B-spline method performs best overall, providing qualitative registrations up to very high deformations as indicated by its flat maximum.

In the first experiment, a large series of *μ*CT scans was acquired continuously with an increasing displacement at a well chosen compression speed to minimize motion artefacts in a single rotation. As such, the registration can be scored as a function of displacement, retrieving the optimal working range of the methods. The scoring curves for the three different registration methods can be observed in Fig. 1, which is obtained by progressively registering the first registration to the reconstruction of a rotation further in time. The value plotted on the abscissa of this figure is the displacement of the compression stage *n*_*v*_ in units of voxels (of 40 *μ*m each). It should be noted that this nominal value is a maximum for the displacement of all the voxels, and the mean displacement is much less. As a reference, this nominal displacement amounts to approximatelyy 3.3 voxels per rotation.

Notably, the curves in Fig. 1 show a maximum, implying that the initial deformations are not large enough for the registrations to significantly boost the NCC, and that the algorithms lose track of deformations that are too large. The maximum should thus be interpreted as the optimal state of deformation at which the registration algorithms are most effective. More importantly, beyond the maximum the registration results are not to be trusted entirely. Indeed, while some regions of the images might be registered correctly, other regions might not be registered or even be distorted by the inferred deformation field.

### Reducing motion artefacts

Having proven its worth as an effective registration method, the B-spline technique is now used to estimate a displacement vector field between the first reconstruction of the aluminium foam acquisition, i.e. the fixed image, and a dataset corresponding to the B-spline scoring curve’s maximum (see Fig. 1), i.e. the moving image, with a varying degree of motion artefacts. By following the motion corrective scheme discussed in the Materials and Methods section, several strategies can be devised to reconstruct the time instances within and even outside of the range bounded by the fixed and moving frames. Let *τ*_*f*_ and *τ*_*m*_ denote the time stamps linked to these respective frames, then any time instant ${\tau }_{S}\in [{\tau }_{f},\,{\tau }_{m}]$ can be reconstructed by selecting a random set *S* of projections in between these two time instances, which at least provides a sufficient angular sampling. Since the exposure time is constant throughout the entire experiment, the time scale may be expressed in terms of projections indices (which is equivalent to *t*_*exp*_ being the unit of time), with *τ*_*f*_ = 350 and *τ*_*m*_ = 10850. Three scenarios are now considered to reconstruct the volume at time instance *τ*_*c*_ = 5600, at the centre of the interval:

- case 1: The volume at time instance *τ*_*c*_ is reconstructed with the 700 projections from its enclosing rotation, which is the standard approach.
- case 2: The volume at time instance *τ*_*c*_ is reconstructed using 700 projections, equidistantly sampled across the interval [*τ*_*f*_, *τ*_*m*_].
- case 3: The volume at time instance *τ*_*c*_ is reconstructed using 2800 projections, which is the equivalent of four complete rotations, though again equidistantly spread out across the interval [*τ*_*f*_, *τ*_*m*_]. Due to the continuous acquisition protocol, the angular sampling is not improved as compared with case 2.

**Figure 2:**
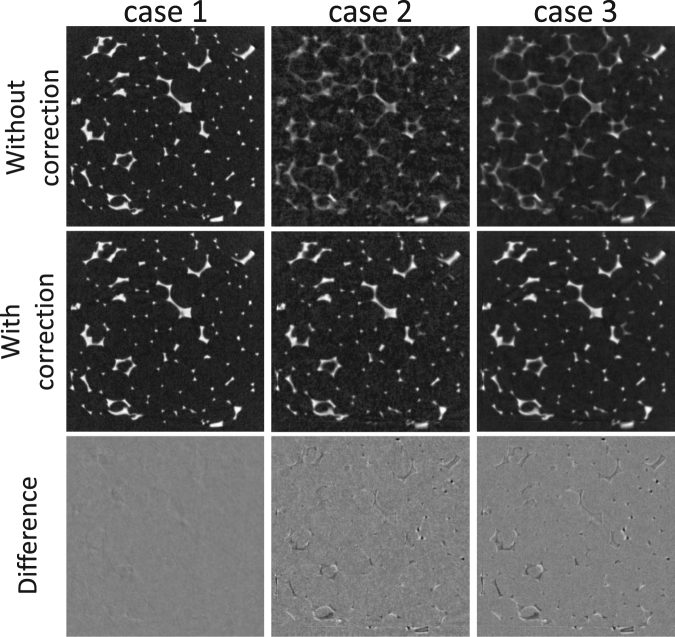
Three cases showing the impact of a motion corrected reconstructions of compressed aluminium foam. From left to right: a standard single rotation reconstruction with 700 projections (case 1), a reconstruction with 700 projections spread over 14 rotations (case 2) and a reconstruction with 2800 projections spread over 14 rotations (case 3). The difference images are referred to the uncorrected reconstruction of case 1. The size of the visualized area is approx. 1.5 × 1.5 cm^2^.

An axial reconstruction slice for each of these three scenarios is shown in Fig. 2, with and without engaging the motion correction scheme. The MVF, used in this correction is set out on a 256^3^ grid, which is decimated by a factor of two with respect to the original 512^3^ reconstruction grid (of 40 *μ*m voxels). By incorporating this vector field, the motion blurring artefacts can effectively be eliminated, which is very clear in the second and third reconstruction case, while in the first case there is practically no motion blurring to begin with. As an important note on case 3, it should be mentioned that the reconstruction is actually performed with an ordered subset approach, i.e. OS instead of SART with four projections in each subset. The OS approach is adopted here to avoid the inherent instability of SART at an excessive amount of iterations. As such, due to this alternate OS approach, the improved SNR in case 3 can not solely be attributed to the incorporation of more projections. Finally, in comparing cases 2 and 3 to the first case, which may serve as a ground truth for reconstruction of *τ*_*c*_, there are some residual motion artefacts, which could not be eliminated through the motion corrected reconstructions.

### Single rotation deblurring

**Figure 3:**
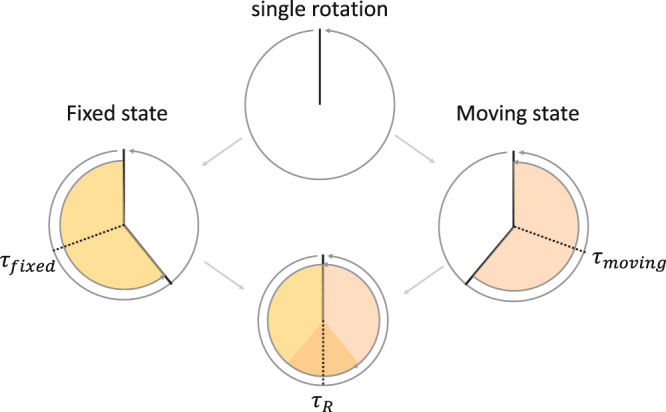
The angular ranges of the sub-reconstructions used for motion correction of a single rotation *μ*CT scan.

In the first experiment, motion artefacts within a single 360 rotation are negligible as the scanning speed is high. However, in many cases the speed is limited by the required signal-to-noise ratio in the reconstruction. This is evaluated in the second experiment, which only contains a single 360 rotation and a varying compression. As such, we aim to estimate a deformation field by strategically defining sub-acquisitions in the acquired datasets, that reflect at least two different states of motion (fixed and moving) of the aluminium foam cube. The logical choice is to assign the fixed and moving frames to the reconstructions of the two short scans, that are separated furthest in time within the bounds of the full rotation, i.e. the angular ranges [0, 180 + 2*γ*] and [180 − 2*γ*, 360] (see Fig. 3), where *γ* denotes the half cone opening angle, indicating the minimum amount of data required to reconstruct a cone-beam dataset^49^. The maximum compression reached in the 16 min scan, is approximately the same as the compression reached up to the maximum of the B-spline scoring curve in the previous experiment, i.e. 1.6 mm versus 1.75 mm, respectively. The 16 min acquisition is thus already situated at the limits of deformation that can be registered with the B-spline technique. Moreover, the fixed and moving images, how they are defined here, are expected to also show a certain degree of motion blurring, which may hinder the registration step.

**Figure 4:**
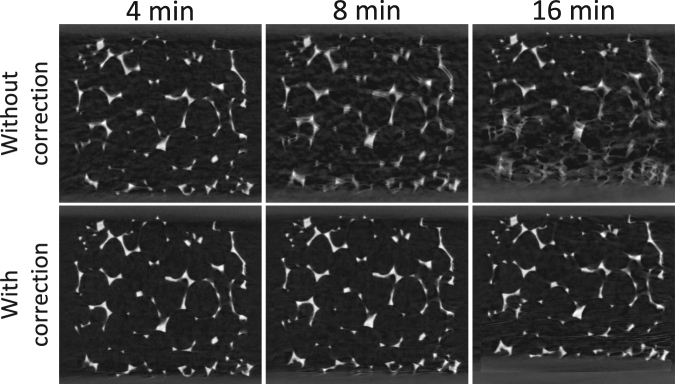
Reconstruction of a single rotation acquisition with and without motion correction by registering the short scan sub-acquisitions. Most of the motion artefacts are eliminated, yet for the 16 min acquisition an aliasing artefact arises. This can be attributed to the bottom plate of the compression stage that gradually enters the registration window. Most registation techniques have difficulties to handle this sudden introduction of extra image features. The size of the visualized area is approx. 1.5 × 1.3 cm^2^.

In Fig. 4, a vertical slice for the motion blurred reconstructions of the 4, 8 and 16 minute acquisitions are compared to their motion corrected versions, where the deformation vector field between the short scan sub-acquisitions is incorporated into the reconstructions. Most of the motion blurring artefacts are eliminated, even in the relatively long 16 min scans. However, new artefacts are introduced in the reconstruction of the 16 min acquisition (corresponding with a very large deformation), as the highly-attenuating bottom plate of the compression stage enters the registration window. The discrepancy between this large displacing feature, and the implicit assumption of zero displacement at the registration window boundaries, leads to a form of temporal aliasing. In general, most registration techniques fail on the image boundaries, especially when matter enters or exits through them. Indeed, to explain the unexpected loss or gain in image intensity and features, boundary sources and sinks should be incorporated into the registration models, a future development that fits into the discussion section.

## Discussion

Purely based on the scores in Fig. 1 alone, the B-spline method is clearly superior to the others, providing robust and qualitative registrations up to very a high deformation (*n*_*v*_ ≈ 100 voxels) as indicated by its flat maximum. Moreover, the B-spline method is also very fast (under 1 minute per registration of a 256^3^ displacement grid), despite the fact that it is inherently more complex, specifically in its higher order resampling steps. This speed is a direct result of the excellent rate at which a set of B-splines can approximate a function, i.e. it can accurately represent (smooth) functions with a relatively small amount of control grid points.

**Figure 5:**
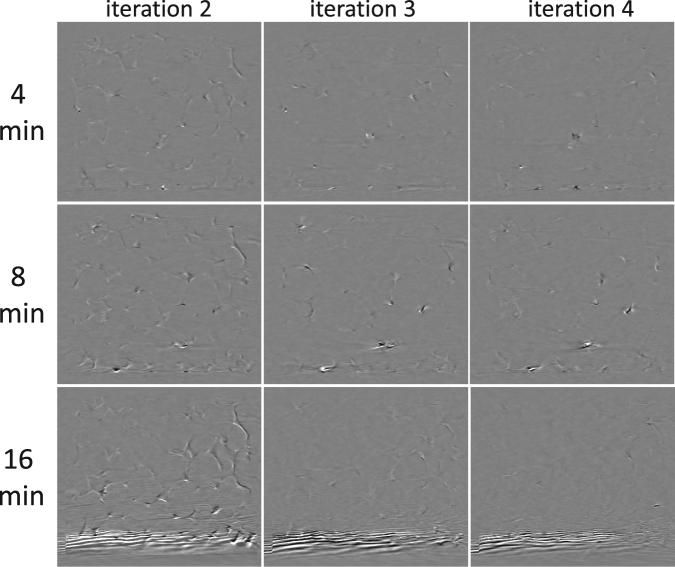
Multiple iterations in a motion corrected reconstruction of a single rotation acquisition. These images show the incremental differences between the results of the current and the previous iterations. By performing multiple rotations the image quality is not significantly improved. Moreover, the temporal aliasing artefacts in the 16 min acquisition are amplified persistently throughout several iterations. The size of the visualized area is approx. 1.5 × 1.3 cm^2^.

In the results of the single rotation motion correction, presented in Fig. 4, only one iteration has been performed. A logical next step is to iterate the motion correction approach to gradually eliminate the motion artefacts in the short scan sub-acquisitions. It is plausible to assume that in doing so, better estimates for the deformation vector field can be found at each iteration. However, the deformation vector field did not significantly change from iteration to iteration, implying that its solution is stuck in a flat optimum. As a result, the image quality also did not improve significantly by performing multiple iterations. On the contrary, the aliasing artefacts introduced through a motion correction of the 16 min scan seemed to be amplified, and persisted throughout further iterations (Fig. 5). Intuitively, the flat nature of the deformation field optimum can be explained by considering that the short scan reconstructions are corrected by deformation vector fields, whose estimates are based on these same reconstructions. In the light of consistency, it is logical that more iterations will not produce widely different deformation vector fields, or at least vector fields that will further promote the motion correction. A future, potential improvement may be found in higher order temporal parametrisations of the vector field, i.e. other than Eqn. (9), by taking into account multiple time instances through a short scan window that slides across the entire acquisition.

Next to their motion corrective potential, registration techniques also show a great potential towards the analysis of a sample’s material characteristics. Indeed, a strain tensor can be mapped across an entire sample through a proper differentiation of the deformation vector field. For example, when the strains are small enough, Cauchy’s strain tensor can be considered. By adopting simplifications such as a linear elastic and isotropic material model, a stress field can be deduced. An example hereof is shown in the supplementary materials (Movie 1), which visualizes the magnitude of the stress vector as a function of time during compression of the aluminium foam. Though this is only an exemplary result which does not yield correct quantitative results given the inelastic deformations of the aluminium foam structure, it illustrates the potential of this framework in materials sciences, where ultimately material properties can be calculated from the comparison between the estimated displacements and FEM simulations based on the images. As such, this framework can provide a large added value in a combination with an in-depth modelling of the deformation itself, e.g. through finite element models. By doing this, X-ray *μ*CT could be elevated from a simple attenuation imaging technique to a way of characterising a sample’s material properties at a micro-structure level.

## Electronic supplementary material

Supplementary material
